# Electroencephalogram Source Imaging and Brain Network Based Natural Grasps Decoding

**DOI:** 10.3389/fnins.2021.797990

**Published:** 2021-11-30

**Authors:** Baoguo Xu, Leying Deng, Dalin Zhang, Muhui Xue, Huijun Li, Hong Zeng, Aiguo Song

**Affiliations:** The State Key Laboratory of Bioelectronics, Jiangsu Key Laboratory of Remote Measurement and Control, School of Instrument Science and Engineering, Southeast University, Nanjing, China

**Keywords:** natural reach-and-grasp decoding, movement-related cortical potential, EEG source imaging, phase locking value, brain network

## Abstract

Studying the decoding process of complex grasping movement is of great significance to the field of motor rehabilitation. This study aims to decode five natural reach-and-grasp types using sources of movement-related cortical potential (MRCP) and investigate their difference in cortical signal characteristics and network structures. Electroencephalogram signals were gathered from 40 channels of eight healthy subjects. In an audio cue-based experiment, subjects were instructed to keep no-movement condition or perform five natural reach-and-grasp movements: palmar, pinch, push, twist and plug. We projected MRCP into source space and used average source amplitudes in 24 regions of interest as classification features. Besides, functional connectivity was calculated using phase locking value. Six-class classification results showed that a similar grand average peak performance of 49.35% can be achieved using source features, with only two-thirds of the number of channel features. Besides, source imaging maps and brain networks presented different patterns between each condition. Grasping pattern analysis indicated that the modules in the execution stage focus more on internal communication than in the planning stage. The former stage was related to the parietal lobe, whereas the latter was associated with the frontal lobe. This study demonstrates the superiority and effectiveness of source imaging technology and reveals the spread mechanism and network structure of five natural reach-and-grasp movements. We believe that our work will contribute to the understanding of the generation mechanism of grasping movement and promote a natural and intuitive control of brain–computer interface.

## Introduction

Brain–computer interface (BCI) is a control system, which enables users to directly communicate with the external environment through electroencephalogram (EEG) (Wolpaw et al., 2000). In many applications of BCI, the field of motor rehabilitation has attracted many researchers’ attention. Moreover, as an essential human skill, hand function is the first choice for many people with dyskinesia want to restore (Snoek et al., 2004). Studies in the early 21st century have shown that neural prosthesis control can be achieved by noninvasive BCI, but mainly depends on simple and repeated imagination of motor tasks (Pfurtscheller et al., 2003; Rohm et al., 2013), which gives an unnatural controlling feeling for BCI users.

To extend the BCI instruction set and realize a natural and intuitive control, many researchers have investigated the possibility of decoding complicated grasping information using movement-related cortical potentials (MR) (Jochumsen et al., 2015; Ofner et al., 2016; Pereira et al., 2017; Shiman et al., 2017). MRCP is considered as a low-frequency EEG negative shift that contains rich movement information, including movement types, force, and speed (Shakeel et al., 2015). Using MRCP, Schwarz et al. (2018) successfully decoded three natural grasp types (palmar, pincer, lateral) in the same limb and no-movement condition with a peak performance of 65.9% and identified their significant differences in MRCP. Ofner et al. (2019) investigated MRCPs of five attempted arm and hand movements, and the peak accuracy of five-class classification was 45%. Moreover, they found discriminative signals originated from central motor areas based on pattern analysis. In our previous study, we investigated five natural grasp types and no-movement condition using MRCP, and five-class classification accuracy was significantly better than significance level (Xu et al., 2021).

However, most of these studies are based on channel features. As we know, limited by the volume conductor effect, the spatial resolution of EEG signals is low, which is not conducive for us to understand how EEG is produced and distributed in the brain. To solve this problem, EEG source imaging (ESI) may be a good solution. Many studies about decoding of hand movement EEG reported that the source-based method outperformed sensor-based method (Yuan et al., 2008; Handiru et al., 2016). The previous study by Edelman et al. (2016) verified that ESI can improve decoding performance when classifying four complex right-hand motor imagery (MI) tasks: the accuracy of individual task classification and overall classification after using ESI was, respectively, 18.6 and 12.7% higher than the peak performance obtained using the sensor-based method. Moreover, a recent study that combined scout ESI and convolutional neural network presented a high classification performance for 4-class MI tasks: the overall accuracy was 14.4% higher than the accuracy achieved using the state of the art MI classification methods (Hou et al., 2020). Because of the proximity of motor brain regions activated by the same limb, we hope to improve spatial resolution using ESI technology and obtain a higher classification result.

Recently, a novel and effective way to investigate the communication patterns of the brain regions has attracted many researchers’ attention, which is brain network analysis. The study of brain network is often described by functional connectivity and graph theory. For example, the recent study by Gu et al. revealed the network distinction between left and right foot MI tasks based on network-based statistics and graph theory indices (Gu et al., 2020). In addition, Xiong et al. (2020) constructed functional connectivity matrices in five frequency bands based on weighted phase lag index and used the network metrics from these matrices to decode different movement intentions. However, few studies focus on constructing brain networks in MRCP frequency band, especially on natural grasping movements.

In our study, we collected EEG signals for five natural grasp types as well as no-movement condition. We applied ESI technology on the MRCP frequency band and used phase locking value (PLV) between the regions of interest (ROIs) to construct functional connectivity matrices. In addition, five network metrics were calculated to show the changes of brain networks of different movements over time. Our study aimed to answer three questions: (1) whether the classification performance can be improved by using source features compared with using channel features; (2) whether the conclusions obtained by using source features are consistent with those obtained by using channel features; and (3) whether there are significant differences in brain networks and network metrics of different grasp types and what changes they show in the whole reach-and-grasp process. We hope our study can shed some light on other researchers.

## Materials and Methods

### Subjects

This study was approved by the ethics committee of Southeast University. Eight right-handed subjects (five female) aged between 22 and 25 years were recruited. All subjects were without any history of neuromuscular disorders and had normal or corrected-to-normal vision. All subjects were accurately explained the purpose and procedure of the experiment and signed the informed consent.

### Experimental Setup

**FIGURE 1:**
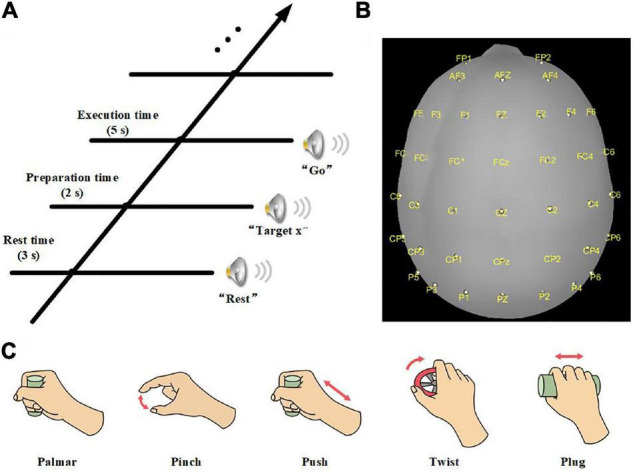
**(A)** Experimental paradigm of one trial. **(B)** Electrode layout. **(C)** Five daily used natural reach-and-grasp movements.

The experiment was carried out in an electromagnetic and noise-shielded room with weak light, good ventilation, and appropriate temperature. We used an audio cue-based paradigm, as shown in Figure 1A. Subjects were instructed to perform six kinds of tasks, which were palmar, pinch, push, twist, plug, and no-movement, as illustrated in Figure 1C, and these tasks appeared in random order. At second 0, an auditory beep instructed subjects to place their right hands on the press button and take a 3-s rest. After that, an auditory cue indicated a specific task, and subjects were asked to stay focused and avoid swallowing or blinking. At second 5, a “go” cue instructed subjects to execute the required movement in 5 s, including reaching, grasping, and returning. For no-movement instructions, subjects should avoid any eye or body movement.

The experiment was divided into 8 sessions, and 10 trials were recorded for each task in a session. Therefore, 480 trials were obtained for each subject. Furthermore, we recorded a 10-s rest at the beginning of the experiment, and an 8-min rest was introduced between sessions to reduce the fatigue of subjects.

### Data Recording

We used a 64-channel active electrode cap (BrainProducts, Germany) and a SynAmps2 amplifier (Neuroscan Compumedics, United States) for EEG recording. The electrodes were arranged following the international standard 10–20 montage. Forty electrodes positioned over frontal, parietal, and temporal lobes were selected to collect the signals (Figure 1B). We used the left mastoid as a reference and the FPz channel as ground. The electrode impedances were kept less than 5 kΩ. EEG was sampled with 1,000 Hz and prefiltered between 0.05 and 100 Hz. Besides, a notch filter at 50 Hz was applied to remove power-line noises. In addition, we used a press button and force transducers to record force information and analyze the reach-and-grasp phase.

### Movement Onset Detection

For detecting the movement onset of each trial, the rising edge of the pressure button was used. Similarly, the falling edge of the pressure button was defined as the end of the movement. For no-movement conditions, we calculated the mean reaction time of movement trials of each subject and added it to the “go” cue onset of no-movement trials. The reaction time was the period between the “go” cue onset and the actual movement onset. In addition, the time when subjects started and finished their grasp was also determined by the rising and falling edge of corresponding force signal. In this step, we discarded the trials that met one of the following four conditions: (1) subject released press button before “go” cue; (2) subject did not return to the press button in 5 s after “go” cue; (3) subject’s reaction time was more than 1.5 s; and (4) subject executed a wrong movement.

### Signal Preprocessing

Signals were processed using MATLAB R2020b and EEGLAB toolbox (Delorme and Makeig, 2004).

First, EEG signals were filtered from 0.1 to 40 Hz using a zero-phase fourth-order Butterworth filter and then downsampled to 100 Hz. Afterward, noisy channels were removed by visual inspection.

To remove stationary artifacts, we high-passed filtered EEG greater than 1 Hz using a zero-phase fourth-order Butterworth filter and computed independent components (ICs) with the extended infomax independent component analysis (ICA) (Lee et al., 1999). In this step, the ICA weights were cached, and contaminated ICs were visually identified and removed with the help of EEGLAB implementation of ADJUST (Mognon et al., 2011) and ICLabel (Pion-Tonachini et al., 2019). After that, the rest ICs were projected back to the channel space and trials with (1) amplitude exceeding ± 100 μV, (2) abnormal joint probability, and (3) abnormal kurtosis were marked for rejection. The threshold of (2) and (3) was five times the standard deviation of their statistic.

Then, we applied the cached ICA weights to the original data (0.1–40 Hz filtered). Previously identified ICs were rejected, and data were back-projected to the channel space. We extracted epochs in the [−2, 3.5] s interval of each trial as time ROI (tROI), with 0 s corresponding to movement onset. Furthermore, trials with rejection marks were excluded.

Lastly, data were converted to a common average reference. We adopted a zero-phase fourth-order Butterworth filter between 0.1 and 3 Hz to get MRCP.

### Electroencephalogram Source Imaging

To make results more explanatory, MRCP were mapped from the channels to the cortical surface through ESI. The ESI processing was based on Matlab Brainstorm (Tadel et al., 2011). Colin27 template boundary element head model was chosen because of the high three-dimensional resolution. We used OpenMEEG (Gramfort et al., 2010) to solve the forward problem and sLORETA (Pascual-Marqui, 2002) to compute sources. To reduce the impact of sensor noise, we used 10-s resting data, which were similarly preprocessed as the movement data, to estimate a noise covariance matrix.

**FIGURE 2:**
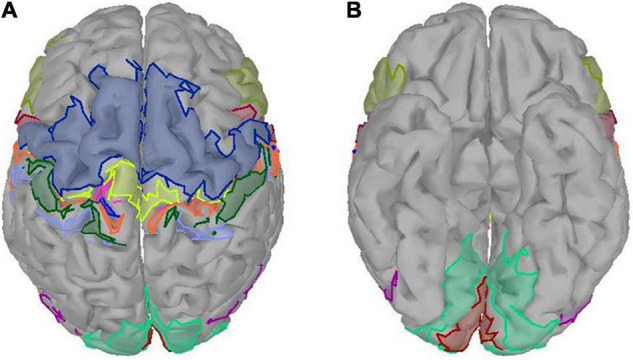
Twenty-four ROIs defined by an atlas of a subset of the Brodmann areas. Different colored regions referred to different ROIs. **(A)** Top view. **(B)** Bottom view.

The ROIs in our study were defined by an atlas of a subset of the Brodmann areas (Fischl et al., 2008). There were 24 ROIs, which covered somatosensory area (BA1, BA2 BA3a, BA3b), primary motor area (BA4a, BA4p), premotor area (BA6), Broca area (BA44, BA45), primary visual area (V1), secondary visual area (V2), and visual area of middle temporal (MT) of both hemispheres (Figure 2). The purpose of studying the visual areas is to investigate whether different grasping objects have significantly different activation patterns on the visual areas. To reduce the computation, we averaged the sources of MRCP in the same ROI and referred to it as sMRCP.

### Feature Extraction and Classification

In our study, we were interested in the six-class classification performance, as in practical application scenarios, BCI equipment should not only distinguish complicated grasp types, but also recognize whether users want to grasp.

The feature extraction method and classifier in our study followed the practice of Schwarz et al. (2018). The difference was that they extracted the amplitude of MRCP as features, while we extracted the amplitude of sMRCP. First, sMRCP was resampled to 16 Hz to ease computational pressure. Next, a 1-s time window was used to take amplitude values in 125-ms steps as features. For classification, we used shrinkage-based linear discriminant analysis classifiers (sLDA) (Blankertz et al., 2011). For every trial, we moved the time window in steps of 1/16 s over the tROI and finally obtained 88 sLDA models over the whole tROI and 192 features for every model. Finally, fivefold cross-validation was repeated 10 times, and the mean accuracy was reported.

To figure out the impact of the volume conductor effect, we compared the classification performance obtained using channel features and source features. We also investigated the impact of time window size on the overall classification performance using four window sizes (one sample, 0.5, 1, and 1.5 s). Furthermore, we calculated precision and confusion matrices at specific time points to see which grasp type had a major impact on the classification performance.

### Brain Network Analysis

To investigate the motion coding process, we calculated functional cortical connectivity using PLV (Lachaux et al., 1999). PLV is a measure of synchronization in the time domain and the single-trial formula definition is as follows:


$$\text{PLV}\left(t\right)=\frac{1}{N}\left|\sum_{n=1}^{N}\text{exp}\left(j\Delta \varphi \left(t\right)\right)\right|$$

where *t* is the specific time point, *N* is the number of sample points, and Δφ(*t*) is the difference of instantaneous phases between pairs of ROIs at time *t*. In this article, instantaneous phases were obtained by computing analytic signal using Hilbert transform, and a sliding window of 1 s with 0.1-s step size was adopted for PLV calculation.

A network is usually represented as a graph that consists of nodes and edges. In our study, nodes were 24 ROIs, and the weight of edges was PLV between pairs of ROIs. To avoid spurious connections, we filtered the edges whose PLV was less than 0.65. Besides, we used Gephi to produce functional connectivity maps (Bastian et al., 2009).

In this study, five network metrics were applied to investigate the trends and properties of networks, which were the mean degree (DE), density (DS), characteristic path length (CPL), clustering coefficient (CC), and modularization (M) (Fornito et al., 2016). DE of a node is the sum of the weight of edges connecting this node with all other nodes, which describes the significance of a node. DS is the ratio of the number of actual connections to the maximum number of possible connections. CPL is the average shortest path length between two nodes in a network. CC can be thought of as the probability of finding a connection between any two neighbors of the node. M can be used to divide nodes into densely connected subgroups called modules, and nodes within these modules are more strongly connected than with other parts of the network. CPL indicates functional integration, whereas CC and M indicate functional segregation.

## Results

### Behavioral Analysis

**FIGURE 3:**
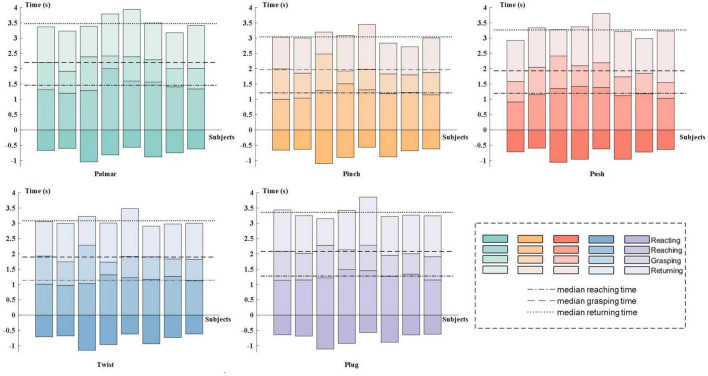
Subject-specific behavioral analysis. In each subgraph, subjects are represented on the horizontal axis in ascending order of subject number from left to right. The 0 s is the subject-specific movement onset. The color tone from dark to light corresponds to the four stages of reach-and-grasp action: reacting phase, reaching phase, grasping phase, and returning phase.

Figure 3 presents the results of behavioral analysis of all subjects for each reach-and-grasp movement. The movement onset and end were defined by the press button, and the grasping phase was specified by force transducers. The 0 s in the figure corresponds to the subject-specific movement onset. As shown in Figure 3, the movement time was different between subjects but keep similar between movement tasks, which pointed out that the more time the subject spent on the palmar task than other subjects, the more time he/she spent on other tasks. For every subject, there was no significant reaction time difference between movement tasks. Besides, we observed similar duration for the reaching phases and returning phases. On average, all subjects completed grasping in 2 s after movement onset. In our study, the pinch-and-twist tasks were completed quicker than the other three tasks, which generally cost 3 s.

### Electroencephalogram Source Imaging

**FIGURE 4:**
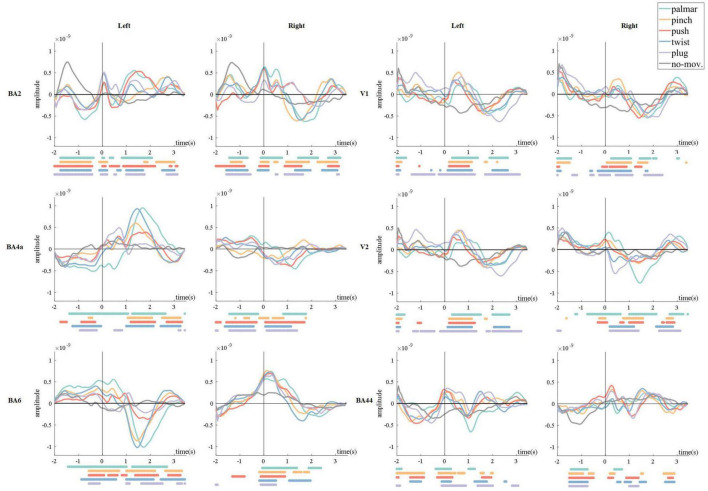
Grand average of all trials of sMRCPs for all conditions. The 0 s corresponds to the movement onset. The dots below each figure were significantly different (*p* < 0.05, Wilcoxon rank sum test) time points between every reach-and-grasp movement and the no-movement.

Figure 4 shows the grand average of all trials of sMRCPs for each condition in BA2, BA4a, BA6, V1, V2, and BA44, corresponding to six brain regions described above.

In Figure 4, we found that in motor-related ROI, for example, BA2, BA4a, and BA6, movement-specific sMRCP in the left hemisphere was more discriminative than that in the right hemisphere, and lateralization could be observed. In addition, we observed a common phenomenon in the left hemisphere of primary motor area (BA4a) and premotor area (BA6): sMRCP often kept steady before 0.7 s and then shifted sharply and reached its peak at approximately 1.5 s, corresponding to the time subjects started grasping. It is worth mentioning that the peak amplitude and peak time of different movement tasks show great distinction. In somatosensory areas, for example, BA2, sMRCP has three peaks, which are at approximately −1.5, 0, and 1 to 1.5 s, respectively. The polarity of these peaks is the same in the left hemisphere, whereas in the right hemisphere, the polarity of the third peak is opposite to that of the first two peaks. Furthermore, the amplitude of the first peak of no-movement is prominently higher than the first peak of movement tasks.

In visual-related ROIs, such as V1 and V2, we did not observe lateralization, but we found significant differences between each grasp type and no-movement condition from 0 to 1 s, corresponding to the reaching phase. Besides, the polarity of sMRCP in the returning phase is opposite to that of the reaching phase. Because of the significant directional sensitivity of the visual areas, we speculated that these phenomena were caused by the object position processing. Moreover, sMRCPs in the left hemisphere of V1 and V2 show a very similar pattern, whereas in the right hemisphere, sMRCP of all conditions in V2 is more discriminative than that in V1 during reaching phase. These phenomena indicated that the same movement activated the left hemisphere of the visual areas in a similar way, whereas in the right hemisphere, secondary visual area (V2) was responsible for more advanced and finer visual processing tasks than the primary visual area (V1).

**FIGURE 5:**
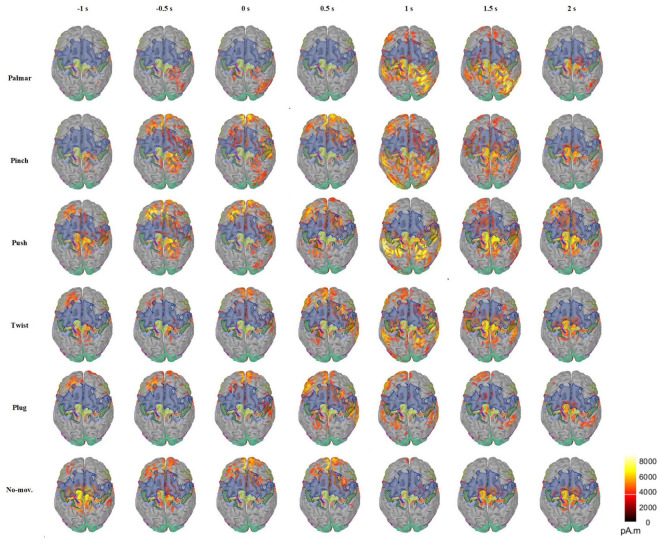
Average cortex maps’ changes for all the conditions of S2 in the time of [−1, 2] s, with 0 s corresponding to the movement onset. In this figure, the activity of sMRCP less than 40% of the maximum activation amplitude was filtered.

Figure 5 presents the changes of sMRCP activity of a representative subject for all conditions in the cortex over time. In Figure 5, we found that grasping movements mainly activated frontal and parietal areas, and the activation patterns were different. In the time of [−1, 0.5] s, the activation of palmar on the cerebral cortex is the least obvious, mainly focusing on the ipsilateral region. In addition, we observed that two groups had similar activation patterns during [−1, 0.5] s: pinch and push, twist, plug, and no-movement. After 0.5 s, the activation starts to spread from the frontal lobe to the parietal and temporal lobes and then shrinks to the central area. Furthermore, we found that the most pronounced activation was at 1 s in the parietal area, except plug and no-movement, corresponding to the time when the subject reached the object and started grasping. At 2 s, we only found obvious activation in the central area, except push. We speculated this was because this time point corresponded to the start of the returning phase under other conditions, but was still grasping phase for push. In addition, Figure 5 also indicated that movement intention activated frontal area most, whereas movement execution mainly activated the parietal area.

### Multiclass Classification Results

**FIGURE 6:**
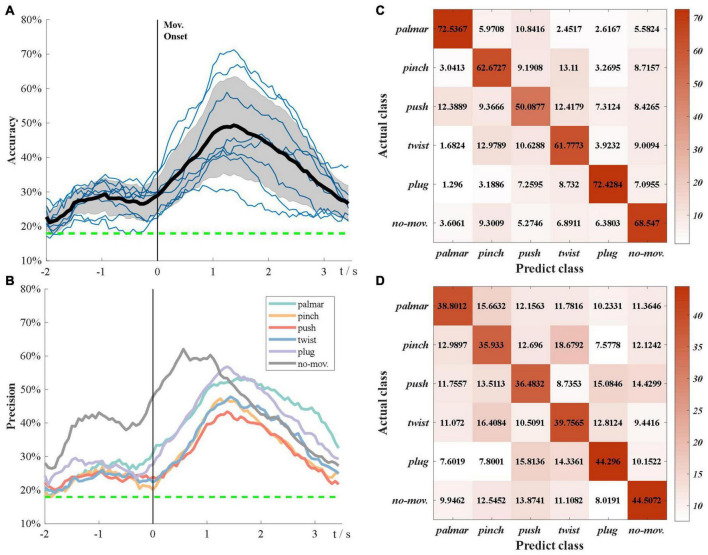
Multiclass classification results. **(A)** Grand average classification accuracy over tROI. The thin blue line represents subject-specific accuracy; the bold line represents grand average accuracy; the shadow shows the standard deviation of grand average accuracy; the green dotted line represents grand average significance level: 17.75%. **(B)** Grand average classification precision of each task over tROI. **(C)** Average confusion matrix of group 1 at grand average peak time. **(D)** Average confusion matrix of group 2 at grand average peak time.

Figure 6A demonstrates the subject-specific and grand average multiclass classification accuracy. The grand average significance level for six-class classification is 17.95% (α = 0.05). In Figure 6A, the grand average accuracy is above significance level during the whole tROI, which not only verifies that sMRCPs can be used to decode movement intentions of complicated grasp types, but also shows excellent performance of source features. In the beginning, the accuracy curves increase slowly until −1 s. After that, there is a temporary decrease followed by a deep increase, which reaches its peak at the grasping phase. We speculated that the temporary decrease was affected by the “go” cue. Compared with the “Target x” cue, “go” cue was a neutral stimulus, which would increase redundant information and lead to the decrease in accuracy.

Figure 6B presents the grand average precision of each task over tROI. All precision curves have similar trends, which are consistent with Figure 6A, whereas the precision of no-movement is significantly higher than that of movement types before the grasping phase. Furthermore, in grasp types, palmar and plug are the easiest to be discriminated, whereas push is the hardest to be identified.

In Figure 6A, we found the classification performance varied among subjects. According to their performance, we divided these subjects into two groups. Group 1 consisted of three subjects with better classification performance: S2, S7, and S8, and the rest subjects were included in group 2. Figures 6C,D present confusion matrices of the two groups at grand average peak time. In both groups, no-movement is easily recognized as pinch, whereas pinch is most likely to be falsely identified as twist, followed by push and no-movement. Group 1 has significantly poor performance in push types, whereas the performance of all types seems more similar in group 2.

Table 1 presents the peak performance obtained using source features and channel features. For some subjects, using source features can achieve better performance, whereas for others, using channel features is better. However, grand average performances are almost the same: 49.35% for source features and 49.65% for channel features. Besides, the reduction of features greatly improves the classification efficiency, which is reflected in the execution time: the single trial execution time of the source-based method is only one-third of that of the channel-based method, which indicates the effectiveness and superiority of source-based method.

**TABLE 1:** Peak performance of using source features and channel features and their corresponding time point.

Subject	Source features		Channel features	
	Accuracy (%)	Time (s)	Accuracy (%)	Time (s)
S1	43.16	1.1875	42.09	1.1250
S2	71.27	1.3750	68.19	1.3125
S3	40.67	1.1875	46.11	1.1250
S4	46.91	1.7500	47.80	1.8125
S5	45.37	1.9375	46.37	1.9375
S6	31.86	0.8125	34.41	0.9375
S7	66.98	1.4375	64.36	1.3750
S8	58.94	1.2500	59.70	1.2500
**Grand average**	**49.35**	**1.3750**	**49.65**	**1.3750**

*The time is relative to movement onset.*

**FIGURE 7:**
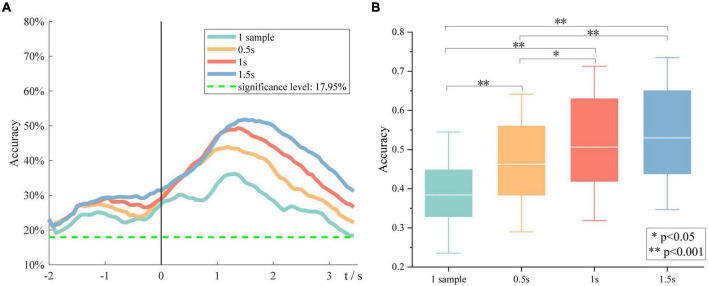
The impact of window size on the overall performance. **(A)** Grand average accuracies of using four window sizes. **(B)** The peak accuracies of all subjects obtained using four window sizes.

Besides, the effect of window size on the overall performance was investigated. Figure 7A demonstrates the grand average six-class classification performance obtained using four time windows, which is one sample, 0.5, 1, and 1.5 s, respectively. We found that with an increase in the window size, the peak accuracy was higher, and the peak time was later. A one-way repeated-measures analysis of variance was performed on the peak accuracies of all subjects at four window sizes. The results are shown in Figure 7B. There is also almost no difference between the performance obtained using a time window of 1 and 1.5 s, but the 1-s window performs significantly better than the 0.5-s window. Therefore, we indicated that the length of source discriminated information was between 1 and 1.5 s.

### Brain Network

**FIGURE 8:**
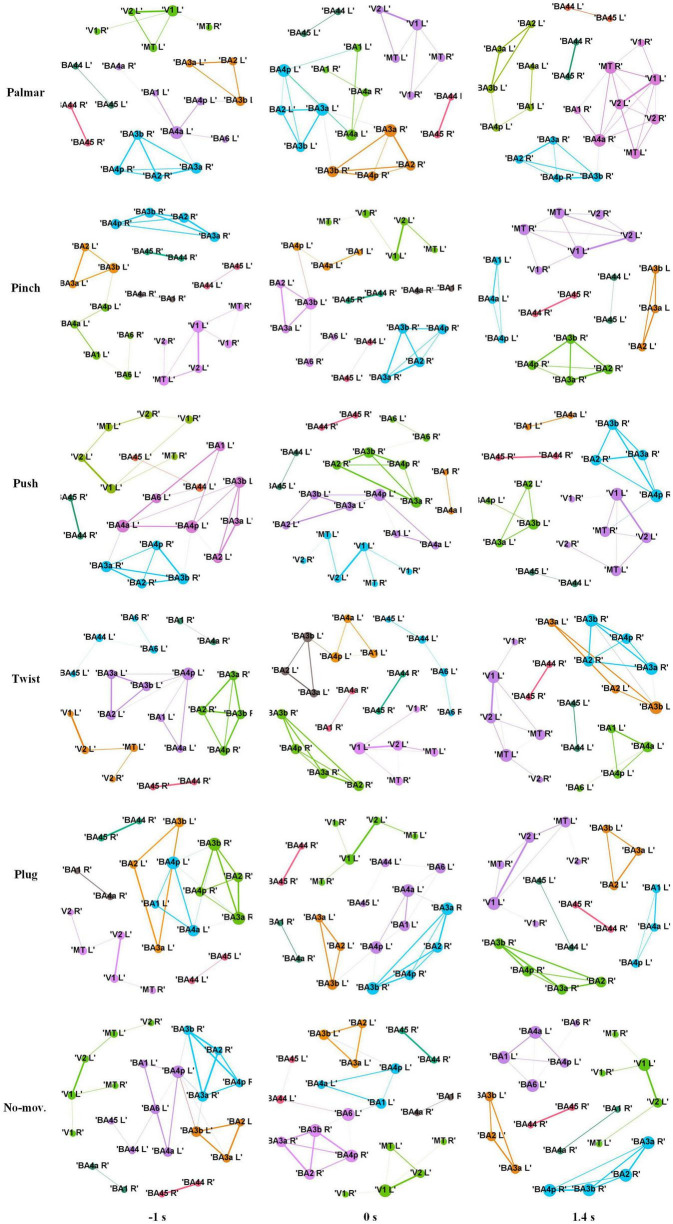
Functional connectivity maps of S2 for each condition. The size of a node indicates DE of the node. The width of an edge indicates the weight of the edge. The edges whose weight is less than 0.65 are filtered. Different colors indicate different modules. Each column represents network structure at different time points, which from left to right are −1, 0, and 1.4 s, respectively.

Figure 8 shows functional connectivity maps of a representative subject (S2) for each condition. Tables 2–6 present DE, DS, M, CC, and CPL of these networks at −1, 0, and 1.4 s, corresponding to the planning phase, movement onset, and peak time. We observed some interesting phenomena from these networks.

**TABLE 2:** The average degree of network for each condition of S2.

Time	Palmar	Pinch	Push	Twist	Plug	No-movement
−1 s	4.19	4	5.333	4.182	4.571	5.271
0 s	5.333	3.652	4.167	4.174	4.545	4.545
1.4 s	6.182	5	4.2	4.762	4.2	4

**TABLE 3:** The density of network for each condition of S2.

Time	Palmar	Pinch	Push	Twist	Plug	No-movement
−1 s	0.21	0.174	0.267	0.199	0.229	0.237
0 s	0.267	0.166	0.181	0.19	0.261	0.216
1.4 s	0.294	0.263	0.221	0.238	0.221	0.2

**TABLE 4:** The modularity of network for each condition of S2.

Time	Palmar	Pinch	Push	Twist	Plug	No-movement
−1 s	0.7	0.753	0.596	0.739	0.601	0.585
0 s	0.61	0.746	0.688	0.772	0.662	0.663
1.4 s	0.594	0.728	0.693	0.7	0.766	0.779

**TABLE 5:** The average clustering coefficient of network for each condition of S2.

Time	Palmar	Pinch	Push	Twist	Plug	No-movement
−1 s	0.667	0.625	0.398	0.671	0.585	0.516
0 s	0.71	0.474	0.538	0.729	0.488	0.644
1.4 s	0.745	0.933	0.682	0.786	0.881	0.744

**TABLE 6:** The average path length of network for each condition of S2.

Time	Palmar	Pinch	Push	Twist	Plug	No-movement
−1 s	2.462	2.154	2.309	1.4	2	2.211
0 s	2.026	2.021	2.429	1.513	2.556	2.778
1.4 s	1.976	1.138	2.222	1.545	1.345	1.419

First, the ROI in the same module is almost always in the same hemisphere. For all these networks, we found only one module that contained ROIs in different hemispheres. This module consists of V1, V2, and MT, representing the visual area.

Second, although the number of modules has a little difference between these networks, which often kept 6, sometimes was 5 or 7, reflected in Table 4, we found (1) both hemispheres of V1, V2, and MT; (2) left hemisphere of BA44 and BA45; (3) right hemisphere of BA44 and BA45; (4) right hemisphere of BA2, BA3a, BA3b, and BA4p; (5) left hemisphere of BA2, BA3a, and BA3b; and (6) left hemisphere of BA1, BA4a, and BA4p are always in the same module in Figure 8. On the one hand, these divisions are consistent with the functions of these ROIs, which are described in the ESI section. On the other hand, groups 4 to 6 show that the connectivity of the left and right hemispheres is asymmetric, and connectivity in the same hemisphere is stronger than that in different hemispheres.

Third, we observed that with the time increased, connectivity between modules decreased, whereas connectivity in modules increased. Tables 5, 6 describe the same phenomenon, where CC increases and CPL decreases over time. This finding indicated that the brain focused more on the communication between modules in the movement intention phase than in the movement execution phase. The work of modules tended to be localized and refined in execution phase.

Lastly, in Tables 2, 3, DE and DS of pinch, push, and twist show a tendency that decreases first and then increases, which is different from palmar, plug, and no-movement: DE and DS of palmar keep increasing over time, whereas le the same properties of plug and no-movement keep decreasing.

## Discussion

In this article, we successfully decoded five natural grasp types and no-movement condition using sMRCP. Multiclass classification results showed that for all subjects, accuracies were better than the grand average significance level during the whole tROI, and a grand average peak accuracy of 49.35% could be achieved at grasping phase. Furthermore, by comparing cortex maps, we found movement intention mainly activated the frontal area, whereas movement execution activated the parietal area. Besides, brain network analysis indicated that modules had more communication in the movement intention phase. During the movement execution phase, the work of modules tended to be localized and refined.

### Multiclass Classification Results

Figure 6A shows that the peak accuracy is achieved at grasping phase. We believed that this was because the shape of hands showed the most significant difference in grasping phase compared with other phases, and this information was also reflected in sMRCP.

Figure 6B demonstrates that the precision of no-movement type is significantly higher than movement types before 1 s and then keeps similar with movement types until the end of the tROI. This phenomenon indicated that significant discriminated information of natural grasp types mainly existed in the grasping phase. In addition, in Figures 6C,D, no-movement is easiest to be recognized as pinch, which may be because pinch uses fewer fingers than other grasp types.

Besides, we found that with the window size becoming larger, the peak performance became higher, and the peak time became later. No significant difference was observed between performances obtained using 1- and 1.5-s time window, indicating that the length of discriminative information in sMRCP was between 1 and 1.5 s. These findings are consistent with the study by Schwarz et al., although their study used channel features (Schwarz et al., 2018).

To find out whether source features performed better than channel features, Table 1 compares the peak performance and the peak time achieved using source features and channel features. Results show that grand average performances are almost the same. It is worth mentioning that the number of source features is less than two-thirds of the number of channel features, indicating that ESI technology is an effective way to improve classification performance. Besides, in Table 1, compared with the results obtained using channel features, subjects with high performance can achieve better performance after using source features, whereas subjects with a poor performance show poorer results. Moreover, the signal quality also had a great influence on the source imaging results: the better the signal quality, the more accurate the source imaging results.

### Grasping Pattern Analysis

Previous studies have shown that the discriminative information of grasp types originated from central motor areas (Ofner et al., 2017, 2019). The same conclusion can be obtained from Figure 5. Besides, we also observed signals of motor areas were more discriminable in the left hemisphere, and this asymmetry was also presented in the number of the network edges in Figure 8. These phenomena reflected the contralateral control of the body by the brain. In addition, we found that the highest distinction of grasp types was in grasping phase in motor and sensorimotor areas. Combined with the research of Sburlea et al. (2021), we speculated that signals in this phase contained not only grasp-type information but also the number of fingers.

Besides, cortex maps in Figure 5 show a pattern that grasping movements mainly activate the frontal-parietal brain networks, which verifies the results in previous studies. Furthermore, in Figure 5, we found that the frontal area was associated with movement intention, whereas the parietal area correlated with movement execution. This is in line with the finding of Sburlea et al. (2021): frontal-central and parietal-occipital activations represent object properties at the planning and execution stage, whereas the primary somatosensory, motor, and parietal areas focus on the processing of grasp types and number of fingers.

In addition, visual area shows a different pattern from motor areas. On the one hand, sMRCPs of motor-related ROIs in Figure 4 presented significant lateralization, but we did not observe the same phenomenon in visual-related ROIs. However, significant differences between each grasp type and no-movement condition were found in the reaching phase. We speculated that visual areas were mainly responsible for processing the object shape and position at this stage. On the other hand, in Figure 8, only visual modules contain ROIs of both hemispheres. For motor modules, ROIs of different hemispheres are divided into different modules. This suggested that connections in motor regions are more regional and localized than in visual regions.

### Limitations and Future Work

Our study proved that multiclass natural grasp types can be discriminated by MRCP in source space, and source features could achieve similar peak performance with channel features. Besides, grasping pattern was also analyzed using cortex maps and network measures. However, our work still has some limitations.

On the one hand, we used the sLORETA to project MRCP into the source level. Although sLORETA is a mature and popular technology for solving the inverse problem, it is hard to obtain the real source as it is an ill-posed problem. Nonetheless, because of the perfect first-order localization of sLORETA, the source obtained in this study is considered effective.

On the other hand, during brain network analysis, we set a threshold of 0.65 for the PLV connectivity matrices for avoiding spurious connections. We were not sure whether using different thresholds could obtain similar conclusions. For future work, we would compare the impact of using different thresholds.

Besides, the EEG data were collected in healthy people. Since spinal cord injured (SCI) people have dyskinesia, the conclusions obtained from the study of healthy subjects are not necessarily applicable to them. In the future, we will verify our findings on SCI subjects.

## Conclusion

In our study, five natural reach-and-grasp movements and no-movement were decoded from source amplitude of MRCPs. Compared with using channel features, similar and satisfactory grand average peak performance can be achieved using ESI technology, with only two-thirds of the channel feature number. Grasping pattern analysis demonstrated that the generation patterns varied among different grasp types. Besides, we found that frontal area was associated with movement intention, whereas parietal area correlated with movement execution. Multiclass classification results proved that hand movements are the most distinguishable at grasping stage. Moreover, during the transition from movement intention to movement execution, the work of modules tended to be localized and refined. These findings will contribute to the understanding of the grasping process and eventually realize a natural and intuitive control of BCI.

## Data Availability Statement

The raw data supporting the conclusions of this article will be made available by the authors, without undue reservation.

## Ethics Statement

The studies involving human participants were reviewed and approved by Ethics Committee of Southeast University. The patients/participants provided their written informed consent to participate in this study.

## Author Contributions

BX and LD designed the study, analyzed the data, and wrote the manuscript. DZ set up the experiment platform. MX performed the experiment. HL, HZ, and AS reviewed and edited the manuscript. All authors read and approved the final manuscript.

## Conflict of Interest

The authors declare that the research was conducted in the absence of any commercial or financial relationships that could be construed as a potential conflict of interest.

## Publisher’s Note

All claims expressed in this article are solely those of the authors and do not necessarily represent those of their affiliated organizations, or those of the publisher, the editors and the reviewers. Any product that may be evaluated in this article, or claim that may be made by its manufacturer, is not guaranteed or endorsed by the publisher.

## References

[B1] Bastian M. Heymann S. Jacomy M. (2009). Gephi: an Open Source Software for Exploring and Manipulating Networks. *Proc. Int. AAAI Conf. Web Soc. Media* 3 361–362.

[B2] Blankertz B. Lemm S. Treder M. Haufe S. Müller K.-R. (2011). Single-trial analysis and classification of ERP components–a tutorial. *NeuroImage* 56 814–825. 10.1016/j.neuroimage.2010.06.048 20600976

[B3] Delorme A. Makeig S. (2004). EEGLAB: an open source toolbox for analysis of single-trial EEG dynamics including independent component analysis. *J. Neurosci. Methods* 134 9–21. 10.1016/j.jneumeth.2003.10.009 15102499

[B4] Edelman B. J. Baxter B. He B. (2016). EEG Source Imaging Enhances the Decoding of Complex Right-Hand Motor Imagery Tasks. *IEEE Trans. Biomed. Eng.* 63 4–14. 10.1109/TBME.2015.2467312 26276986 PMC4716869

[B5] Fischl B. Rajendran N. Busa E. Augustinack J. Hinds O. Yeo B. T. T. (2008). Cortical folding patterns and predicting cytoarchitecture. *Cereb. Cortex* 18 1973–1980. 10.1093/cercor/bhm225 18079129 PMC2474454

[B6] Fornito A. Zalesky A. Bullmore E. T. (2016). *Fundamentals Of Brain Network Analysis.* Amsterdam: Academic Press.

[B7] Gramfort A. Papadopoulo T. Olivi E. Clerc M. (2010). OpenMEEG: opensource software for quasistatic bioelectromagnetics. *Biomed. Eng. Online* 9:45. 10.1186/1475-925X-9-45 20819204 PMC2949879

[B8] Gu L. Yu Z. Ma T. Wang H. Li Z. Fan H. (2020). EEG-based Classification of Lower Limb Motor Imagery with Brain Network Analysis. *Neuroscience* 436 93–109. 10.1016/j.neuroscience.2020.04.006 32283182

[B9] Handiru V. S. Vinod A. P. Guan C. (2016). Multi-direction hand movement classification using EEG-based source space analysis. *Annu. Int. Conf. IEEE Eng. Med. Biol. Soc.* 2016 4551–4554. 10.1109/EMBC.2016.7591740 28269289

[B10] Hou Y. Zhou L. Jia S. Lun X. (2020). A novel approach of decoding EEG four-class motor imagery tasks via scout ESI and CNN. *J. Neural Eng.* 17:16048. 10.1088/1741-2552/ab4af6 31585454

[B11] Jochumsen M. Niazi I. K. Taylor D. Farina D. Dremstrup K. (2015). Detecting and classifying movement-related cortical potentials associated with hand movements in healthy subjects and stroke patients from single-electrode, single-trial EEG. *J. Neural Eng.* 12:56013. 10.1088/1741-2560/12/5/056013 26305233

[B12] Lachaux J.-P. Rodriguez E. Martinerie J. Varela F. J. (1999). Measuring phase synchrony in brain signals. *Hum. Brain Mapp.* 8 194–208. 10.1002/(sici)1097-0193(1999)8:4<194::aid-hbm4>3.0.co;2-c 10619414 PMC6873296

[B13] Lee T. W. Girolami M. Sejnowski T. J. (1999). Independent component analysis using an extended infomax algorithm for mixed subgaussian and supergaussian sources. *Neural Comput.* 11 417–441. 10.1162/089976699300016719 9950738

[B14] Mognon A. Jovicich J. Bruzzone L. Buiatti M. (2011). ADJUST: an automatic EEG artifact detector based on the joint use of spatial and temporal features. *Psychophysiology* 48 229–240. 10.1111/j.1469-8986.2010.01061.x 20636297

[B15] Ofner P. Schwarz A. Pereira J. Müller-Putz G. (2016). “Movements of the same upper limb can be classified from low-frequency time-domain EEG signals,” in *Proceedings of the Sixth International Brain-Computer Interface Meeting: BCI Past, Present, and Future*, (Austria: Verlag der Technischen Universität Graz), 69.

[B16] Ofner P. Schwarz A. Pereira J. Müller-Putz G. R. (2017). Upper limb movements can be decoded from the time-domain of low-frequency EEG. *PLoS One* 12:e0182578. 10.1371/journal.pone.0182578 28797109 PMC5552335

[B17] Ofner P. Schwarz A. Pereira J. Wyss D. Wildburger R. Müller-Putz G. R. (2019). Attempted Arm and Hand Movements can be Decoded from Low-Frequency EEG from Persons with Spinal Cord Injury. *Sci. Rep.* 9:7134. 10.1038/s41598-019-43594-9 31073142 PMC6509331

[B18] Pascual-Marqui R. D. (2002). Standardized low-resolution brain electromagnetic tomography (sLORETA): technical details. *Methods Find. Exp. Clin. Pharmacol.* 24 5–12. 12575463

[B19] Pereira J. Ofner P. Schwarz A. Sburlea A. I. Müller-Putz G. R. (2017). EEG neural correlates of goal-directed movement intention. *NeuroImage* 149 129–140. 10.1016/j.neuroimage.2017.01.030 28131888 PMC5387183

[B20] Pfurtscheller G. Müller G. R. Pfurtscheller J. Gerner H. J. Rupp R. (2003). ‘Thought’ – control of functional electrical stimulation to restore hand grasp in a patient with tetraplegia. *Neurosci. Lett.* 351 33–36. 10.1016/S0304-3940(03)00947-9 14550907

[B21] Pion-Tonachini L. Kreutz-Delgado K. Makeig S. (2019). ICLabel: an automated electroencephalographic independent component classifier, dataset, and website. *NeuroImage* 198 181–197. 10.1016/j.neuroimage.2019.05.026 31103785 PMC6592775

[B22] Rohm M. Schneiders M. Müller C. Kreilinger A. Kaiser V. Müller-Putz G. R. (2013). Hybrid brain-computer interfaces and hybrid neuroprostheses for restoration of upper limb functions in individuals with high-level spinal cord injury. *Artif. Intell. Med.* 59 133–142. 10.1016/j.artmed.2013.07.004 24064256

[B23] Sburlea A. I. Wilding M. Müller-Putz G. R. (2021). Disentangling human grasping type from the object’s intrinsic properties using low-frequency EEG signals. *Neuroimage Rep.* 1:100012. 10.1016/j.ynirp.2021.100012 PMC12172748 40567869

[B24] Schwarz A. Ofner P. Pereira J. Sburlea A. I. Müller-Putz G. R. (2018). Decoding natural reach-and-grasp actions from human EEG. *J. Neural Eng.* 15:16005. 10.1088/1741-2552/aa8911 28853420

[B25] Shakeel A. Navid M. S. Anwar M. N. Mazhar S. Jochumsen M. Niazi I. K. (2015). A Review of Techniques for Detection of Movement Intention Using Movement-Related Cortical Potentials. *Comput. Math. Methods Med.* 2015:346217. 10.1155/2015/346217 26881008 PMC4735988

[B26] Shiman F. López-Larraz E. Sarasola-Sanz A. Irastorza-Landa N. Spüler M. Birbaumer N. (2017). Classification of different reaching movements from the same limb using EEG. *J. Neural Eng.* 14:46018. 10.1088/1741-2552/aa70d2 28467325

[B27] Snoek G. J. IJzerman M. J. Hermens H. J. Maxwell D. Biering-Sorensen F. (2004). Survey of the needs of patients with spinal cord injury: impact and priority for improvement in hand function in tetraplegics. *Spinal Cord* 42 526–532. 10.1038/sj.sc.3101638 15224087

[B28] Tadel F. Baillet S. Mosher J. C. Pantazis D. Leahy R. M. (2011). Brainstorm: a user-friendly application for MEG/EEG analysis. *Comput. Intell. Neurosci.* 2011:879716. 10.1155/2011/879716 21584256 PMC3090754

[B29] Wolpaw J. R. Birbaumer N. Heetderks W. J. McFarland D. J. Peckham P. H. Schalk G. (2000). Brain-computer interface technology: a review of the first international meeting. *IEEE Trans. Rehabil. Eng.* 8 164–173. 10.1109/TRE.2000.847807 10896178

[B30] Xiong X. Yu Z. Ma T. Luo N. Wang H. Lu X. (2020). Weighted Brain Network Metrics for Decoding Action Intention Understanding Based on EEG. *Front. Hum. Neurosci.* 14:232. 10.3389/fnhum.2020.00232 32714168 PMC7343772

[B31] Xu B. Zhang D. Wang Y. Deng L. Wang X. Wu C. (2021). Decoding Different Reach-and-Grasp Movements Using Noninvasive Electroencephalogram. *Front. Neurosci.* 15:1206. 10.3389/fnins.2021.684547 34650398 PMC8505714

[B32] Yuan H. Doud A. Gururajan A. He B. (2008). Cortical imaging of event-related (de)synchronization during online control of brain-computer interface using minimum-norm estimates in frequency domain. *IEEE Trans. Neural Syst. Rehabil. Eng.* 16 425–431. 10.1109/TNSRE.2008.2003384 18990646 PMC2597339

